# 

**Published:** 1888-11-03

**Authors:** 


CONTENTS.

**"The Empire of Childhood"** - 65

**Scotch Asylums** - 66

**The Story of the London Fever Hospital** - 67

**The Doctor's Pulpit.**—The Seven Ages of Man—V. The Justice - 69

**In and Out Among the Hospitals.**—By a SECRETARY OF TEN YEARS' STANDING - 70

**The Student's Column** - 70

**Brine Baths for Rheumatism.**—By a SUFFERER - 71

**Notes and News** - 72

**Everybody's Page** - 74

**Annotations.**—Dangerous Ointments.—Women and their Victims.—A Lover's Choice - 75

**Voyages in Vacation.**—VI. The Advantages of Sea Travel - 76

**False Impressions.** By CAROLINE FOTHERGILL, Author of "An Enthusiast," "A Voice in the Wilderness," etc. - 77

**"Discourses to Women on Medical Subjects."**—By a CONSULTING PHYSICIAN - 79

**Scraps and Gleanings** - 80

**Amusements and Relaxation** - 80

EXTRA SUPPLEMENT.—THE NURSING MIRROR.

**En Passant.**—Birmingham Infirmary.—Registration of Midwives.—Nurses and Presents.—Brightening Hospital Life.—Proper Rooms for Nurses.—Born Nurses - xvii
**Letters from a Probationer.**—No. V. Operation Day - xviii
**Opening of a Nursing Institution at Taunton** - xviii
**Presentation** - xviii
**Everybody's Opinion.**—The British Nurses' Association.—Zenana Missions.—Massage.—Army Nursing - xix
**Vacancies** - xix
**Notes and Queries** - xix

